# Applying Fuzzy Logic to Comparative Distribution Modelling: A Case Study with Two Sympatric Amphibians

**DOI:** 10.1100/2012/428206

**Published:** 2012-05-02

**Authors:** A. Márcia Barbosa, Raimundo Real

**Affiliations:** ^1^Rui Nabeiro Biodiversity Chair, Centro de Investigação em Biodiversidade e Recursos Genéticos (CIBIO), Universidade de Évora, 7004-516 Évora, Portugal; ^2^Division of Ecology and Evolution, Imperial College London, Silwood Park Campus, Ascot SL5 7PY, UK; ^3^Laboratorio de Biogeografía, Diversidad y Conservación, Departamento de Biología Animal, Facultad de Ciencias, Universidad de Málaga, 29071 Málaga, Spain

## Abstract

We modelled the distributions of two toads (*Bufo bufo* and *Epidalea calamita*) in the Iberian Peninsula using the favourability function, which makes predictions directly comparable for different species and allows fuzzy logic operations to relate different models. The fuzzy intersection between individual models, representing favourability for the presence of both species simultaneously, was compared with another favourability model built on the presences shared by both species. The fuzzy union between individual models, representing favourability for the presence of any of the two species, was compared with another favourability model based on the presences of either or both of them. The fuzzy intersections between favourability for each species and the complementary of favourability for the other (corresponding to the logical operation “A and not B”) were compared with models of exclusive presence of one species versus the exclusive presence of the other. The results of modelling combined species data were highly similar to those of fuzzy logic operations between individual models, proving fuzzy logic and the favourability function valuable for comparative distribution modelling. We highlight several advantages of fuzzy logic over other forms of combining distribution models, including the possibility to combine multiple species models for management and conservation planning.

## 1. Introduction

Comparative distribution modelling (i.e., building models that combine or compare the distributions of different species) is a useful tool to assess differences and similarities between species' distribution areas and environmental correlates. It has been applied, for example, to species with partially overlapping distributions [1], genetically differentiated subspecific forms [2], cryptic species whose distribution data are difficult to assign [3, 4], and species linked by close biotic interactions [5].

Comparative modelling has mostly been done in pairs, by regressing presences of one taxon against presences of the other [1–4]. However, this poses clear limitations to the modelling procedure: sample size may become considerably smaller than the whole study sample, because only localities with presence of either one or the other taxon (not sites where both are either present or absent) can be used, and only two taxa can be directly compared at a time.

Relatively recent developments in distribution modelling [6] provided tools to obtain environmental favourability values that can be directly compared among species, even when these have different prevalence (i.e., proportion of presences) within the study area. Environmental favourability models have the additional advantage of allowing operations of fuzzy logic (a form of multivalued logic where the truth value may range in degree between 0 and 1) between the predictions for different species [6], opening a range of possibilities for comparative distribution modelling [5, 7].

In this paper, we test fuzzy logic operations as a tool in comparative modelling using two amphibians with partially overlapping distributions, the common toad (*Bufo bufo*) and the natterjack toad (*Epidalea calamita*, formerly *Bufo calamita*), in the Iberian Peninsula (SW Europe). Both species have widespread distributions in the study area, but with local differences that have been related to macroenvironmental factors [1, 8]. We modelled the Iberian distributions of these species, both individually and in different combinations, and then compared the results of these combination models with those of fuzzy logic operations between the two initial individual models. We illustrate and discuss the applicability of fuzzy logic in comparative distribution modelling.

## 2. Materials and Methods

**Figure 1:**
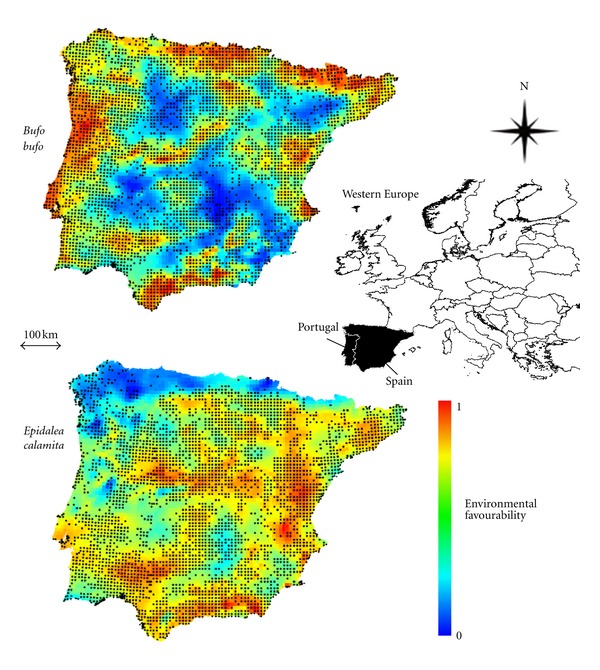
Location of the study area, recorded distributions (black dots: presences on UTM 10 × 10 km squares, after Loureiro et al. [10] for Portugal and Pleguezuelos et al. [1] for Spain), and environmental favourability values predicted for *Bufo bufo* and *Epidalea calamita* across the Iberian Peninsula.

The study area was the Iberian Peninsula, at the south-western edge of Europe (Figure 1). It is a nearly 600,000 km^2^ heterogeneous region comprising the mainland territories of Portugal and Spain and linked to the continent by a narrow and mountainous isthmus. It thus constitutes a discrete biogeographical unit appropriate for studies on species distributions [5, 9].

Species distribution data, consisting of presences and absences on Universal Transverse Mercator (UTM) 10 × 10 km grid cells (Figure 1), were taken from the herpetological atlases of Portugal [10] and Spain [11] and were collected in a roughly similar way. Although some of the absences may result from insufficient surveying effort (false absences), many others are due to ecological or historical reasons, all of which are relevant factors in biogeography. As long as false absences are not spatially structured due to geographically biased sampling effort, they do not reduce model reliability [12]. In any case, false absences are the same as missing true presences, so they affect presence-only models as well.

The UTM 10 × 10 km grid and the limits of the study area were downloaded from the EDIT Geoplatform [13]. We used Quantum GIS 1.7 [14] and its GRASS (Geographic Resources Analysis Support System) plugin [15] to clip the grids with the limits of the study area. Predictor variables, representative of physiography, climate, and human activity (Table 1), were digitized and interpolated in previous studies [16, 17]. We corrected the values of solar radiation [18]. Data management and statistical analyses were carried out in R 2.11 [19] except where otherwise stated. 

We built generalized linear models with a binomial distribution and the logit link of the favourability function [6], which may be written as follows:

(1) $$F=\frac{e^{y}}{n_{1}/n_{0}+e^{y}},$$

where *F* is predicted favourability, *n*_1_ and *n*_0_ are the numbers of presences and absences, respectively, *e* is the basis of the natural logarithm, and *y* is a logit function combining several variables and obtained using logistic regression. Basically, it is a generalized linear model that assesses the local variations in presence probability with respect to the overall species prevalence. This makes the models independent of the species' presence/absence ratio in the study area, enabling direct model comparison and combination when more than one species are involved [5, 7]. 

To avoid a spurious effect of surface area on the probability of the species being present, only complete UTM cells, and not those that are cut by the study area borders or the unions between UTM zones, were used for the inductive stage of the modelling. Models were then applied to the whole study area [5, 17]. 

Variables were included in the models using a forward-backward stepwise procedure [4, 20, 21]. Stepwise selection is a useful and effective tool to infer distribution patterns inductively from observed data, when no theory or previous hypotheses exist about the importance of each variable [5, 22]. Variable selection was based on Akaike's Information Criterion (AIC [23]), and we checked that the same models were obtained when using AIC corrected for large numbers of predictors relative to sample size (AICc [24]). In case any nonsignificant variables remained in a model after AIC-based selection, the model was further updated by removing them step by step, starting with the least significant variable [25]. The following models were built:

(A) a favourability model for *B. bufo*, with 1 = presence and 0 = absence of this species as target data,
(B) a favourability model for *E. calamita*, with 1 = presence and 0 = absence,
(C1) a favourability model for the occurrence of both species together, where 1 = presence of both and 0 = absence of at least one of them,
(D1) a model of favourability for either of the two species, where 1 = presence of at least one and 0 = absence of both species,
(E1) a model of favourability for the presence of *B. bufo* instead of *E. calamita*, where 1 = presence of *B. bufo* only, 0 = presence of *E. calamita* only, and cells where both species are either present or absent were left out of the analysis,
(F1) a model of favourability for the presence of *E. calamita* instead of *B. bufo*, where 1 = presence of *E. calamita* only, 0 = presence of *B. bufo* only, and cells where both species are either present or absent were left out.

Models C1 to F1 were compared, respectively, with their fuzzy logic counterparts from C2 to F2, resulting from the following operations between models A and B:

(C2) fuzzy intersection between the individual models (logic “A and B”),
(D2) fuzzy union of the individual models (logic “A or B”),
(E2) fuzzy intersection between model A and the complementary of model B (logic “A and (not B)”),
(F2) fuzzy intersection between model B and the complementary of model A (logic “B and (not A)”).

Note that models E1 and F1, which use presence-only data, are bound to be the same with contrary signs of the variables' coefficients, but their counterparts E2 and F2 will probably be different. This is why we built both models. 

The capacity of each model to discriminate between the modelled events (i.e., presence versus absence or presence of one species versus presence of the other) was assessed with the Area Under the receiver operating characteristic (ROC) Curve (AUC). This is a widely used model evaluation measure that provides a single-number discrimination measure across all possible classification thresholds for each model, thus avoiding the subjective selection of one threshold [26]. We must keep in mind that, as any discrimination measure, the AUC depends on thresholds (just not on one particular threshold) to convert continuous model predictions into binary classifications, and is strongly conditioned by species prevalence or relative occurrence area [27]. However, this does not affect our pair wise comparisons between models based on combined distribution data and those based on fuzzy logic operations, as the set of data used to assess the AUC is the same in each comparison. 

We also compared the favourability values predicted by the models of combined species data and the corresponding fuzzy logic operations between individual species models, using two different measures: Spearman's nonparametric rank correlation between favourability values, with Dutilleul's [28] sample size adjustment for spatial autocorrelation, implemented in the SAM software [29], and the average overall similarity between maps, calculated with the Map Comparison Kit 3.2.2 (Geonamica/RIKS, The Netherlands), which performs pattern recognition considering logical coherence, local and global similarities [30]. As predictions were numerical, we used the fuzzy numerical comparison, which considers fuzziness of location (the notion that the representation of a cell depends on the cell itself and, to a lesser extent, also the cells in its neighbourhood) in the same manner as the Fuzzy Kappa [29] but is applied to numerical maps, without using a categorical similarity matrix. The following formula was employed to find the fuzzy similarity (FS) of two values *a* and *b* [31]:

(2) $${\text{FS}}_{\left(a,b\right)}=1-\frac{\left|a-b\right|}{\max\left(\left|a\right|,\left|b\right|\right)}.$$

We used the default values for neighbourhood radius and decay, although we tried also a few different values to check that the results were robust.

## 3. Results

**Figure 2:**
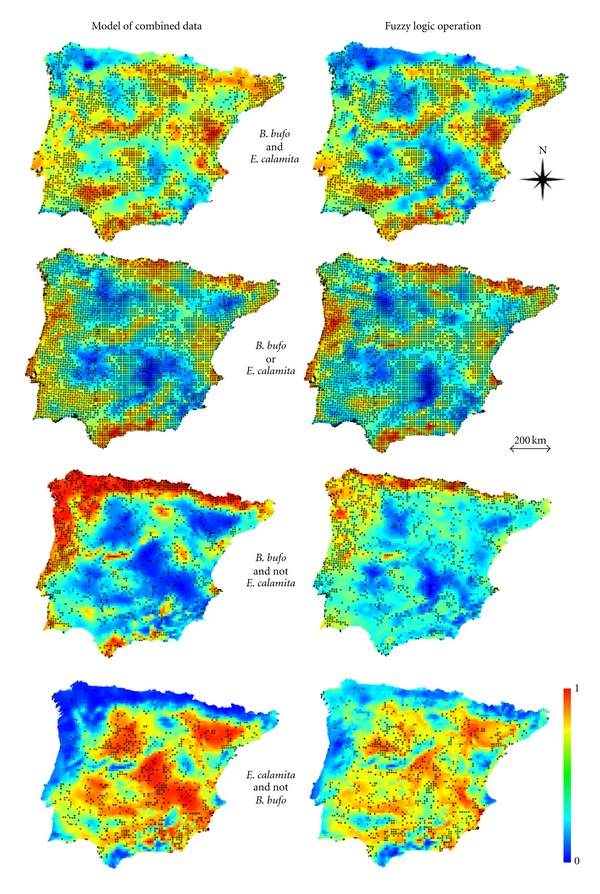
Comparison of predicted environmental favourability for *Bufo bufo* and *Epidalea calamita* given by the models of combined presence/absence data and by fuzzy logic operations between the individual species models. Distribution data (black dots: presences on UTM 10 × 10 km squares) combined from Loureiro et al. [10] for Portugal and from Pleguezuelos et al. [11] for Spain.

There were 3554 presences of *B. bufo* and 3131 presences of *E. calamita *(Figure 1) in the 5464 complete UTM cells used for building models A to D (see also Figure 2 for the distribution of the presences of both species together and the presences of either of the two species). For models E and F, based on complete UTM cells where one and only one of the two species was present, the number of analysed cases dropped to 1861. The ratios between the compared events varied among models (Table 2). 

The individual models obtained for *B. bufo* and *E. calamita* reflect some areas of general agreement between environmental favourability for the two species, in line with the substantial overlap in their distributions; however, there are also areas of disagreement, where one of the two species is clearly more favoured than the other (Figures 1 and 2). The variables included in the models, their coefficient estimates and associated statistics are shown in the Appendix. 

**Figure 3:**
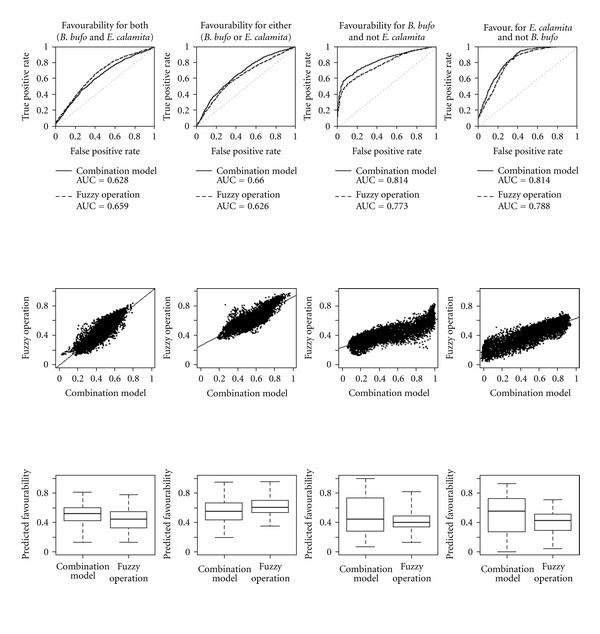
Top row: Comparison of the receiver operating characteristic (ROC) curves and the areas under them (AUC) for models of combined species data and the corresponding fuzzy logic operations between individual species models. Middle row: Scatter plots and linear regression lines comparing favourability values given by combined models and those given by fuzzy logic operations between individual species models. Bottom row: Box plots showing median, upper, and lower quartiles, and extreme values for favourability given by combination models and the corresponding fuzzy operations.

The *B. bufo* model had an AUC of 0.711, while the *E. calamita* model scored a slightly lower 0.629. Spatial autocorrelation in model residuals was negligible (maximum absolute Moran's I was 0.003 for *B. bufo* and 0.002 for *E. calamita*). The models of combined species data and the corresponding fuzzy logic operations between individual species models produced similarly shaped ROC curves and largely overlapping AUC in all four comparisons (Figure 3(a)). 

The predicted values derived from modelling combined species distribution data were also generally similar to the results of fuzzy logic operations between the two single-species models (Figure 2). The similarity between these map pairs is also attested, in all four cases, by both rank correlation and fuzzy numerical comparison of predicted values (Table 2 and Figure 3(b)). For the models of presence of one species against the other, fuzzy logic operations generated less dispersed predictions, with a smaller variation interval (Figure 3(c)).

## 4. Discussion

The relatively low AUC values obtained for both *B. bufo* and *E. calamita* are in line with those generally obtained for species with widespread distributions in the study area [5], as the AUC is known to correlate negatively with species prevalence [27]. Expanding the study area to include the complete distributions of both species could allow obtaining models with larger AUC. However, this would require distribution data at the same resolution from the rest of the distribution areas of both species, which are not available. In addition, higher AUC values do not necessarily mean better calibrated models; they simply reflect the fact that the modelled species does not distinguish so clearly between “good” and “bad” habitat within the studied region. Moreover, as we have pointed out before, this does not affect the pair wise model comparisons, which were the focus of this paper. 

Models confronting the presence of *B. bufo* and *E. calamita* have been built previously, on a narrower spatial scale, in Southern Spain [1]. Analogous models have also been built for other amphibian pairs, such as cryptic species of frogs (*Discoglossus galganoi* and *D. jeanneae* [3]) and newts (*Triturus marmoratus* and *T. pygmaeus* [4]) and genetically differentiated forms of a salamander (*Chioglossa lusitanica* [2]). This may be the adequate approach when the aim of modelling is to assess which environmental parameters distinguish the distribution areas of two organisms. But when the prediction of their potential distributions is the main aim, fuzzy logic operations between the single-species models may be preferable, as they present a series of advantages.

(1) They avoid the need to build additional models: the single-species models are enough.
(2) They allow using all distribution data available, that is, all the localities in the study area, and not only those with exclusive presence of one of the species. This increase in sample size allows a better model calibration and thus can enhance the predictive power of the models.
(3) They allow the possibility of simultaneous multispecies comparisons, instead of comparing species only two by two; models such as C1 may be impracticable when applied to many species, as the number of localities where all the species have been recorded decreases with the number of species analysed, whereas models such a C2 are not affected by this.
(4) Modelling the presence of any of two species (as in model D1 in our study) gives greater weight to the species with higher number of presences, while combining individual species models with fuzzy logic gives the same importance to all species involved.

Our results showed that favourability models for two species combined by means of fuzzy logic operations perform similarly to models of combined data for these species. Although we have not tested this specifically, we may assume that the method will work in other situations, differing, for example, in number of species, the magnitude of the differences between their distribution areas, species prevalence, or the geographical extent of the study area. The modelling method, however, should provide directly comparable numerical predictions, as is the case with the favourability function [6]. 

A fuzzy classification technique (fuzzy envelope model, FEM) has been applied [32] for predicting species' distributions by using presence-only records, although recognizing that when absence records are available, models built using presence-absence data may perform better than presence-only models. In any case, our conclusions are likely applicable to the use of fuzzy logic operations to their fuzzy models, although this needs to be specifically tested. 

Favourability values are here considered as the degree of membership to the fuzzy set of localities favourable to the analysed event (presence of one species, of any of them, of both together, and of one instead of the other). Degrees of membership are sometimes confused with probability values, in part because both take values between 0 and 1. However, the conceptual consequences of this difference between degree of membership and probability are relevant. Local favourability denotes a measure of the degree to which local conditions cause local probability to differ from the probability expected at random, that is, from that expected according to the prevalence of the event [6]. Consequently, favourability values should not be taken as probability values independent of sample prevalence. Local probability depends both on the response of the analysed event to the predictors and on the prevalence of the event [33], whereas favourability depends only on the response to the predictors in the study area [6]. Thus, favourability is aimed at complementing probability, by providing a comparable measure of the response of the event to the predictors for events differing in prevalence. 

The mathematical consequences of this difference between degree of membership and probability are also relevant. The probability of simultaneous occurrence of several events is calculated by multiplying the individual probabilities of each event, which inevitably yields increasingly lower output values as more events are taken into account. The use of fuzzy logic operations avoids this mathematical problem, as favourability for the simultaneous occurrence of several events is computed as the favourability for the least favourable event [34]. This is important when the aim is to identify areas that are simultaneously favourable for groups of several species, as it is the case, for example, in the identification of favourability hotspots [7]. This is especially relevant at a time when distribution modelling of multiple species is increasingly necessary to design effective conservation strategies for both present and future scenarios.

## Figures and Tables

**Table 1:** Factors and their related variables used to model the distributions of *Bufo bufo*, *Epidalea calamita*, and the combined presences of the two species. Sources: ^(1)^U. S. Geological Survey (1996); ^(2)^Font (1983, 2000); ^(3)^I.G.N. (1999); data on the number of inhabitants of urban centres taken from Enciclopédia Universal (http://www.universal.pt) for Portugal and from the Instituto Nacional de Estadística (http://www.ine.es/) for Spain, both in 1999.

Factor	Variable	Code
Topography	Mean altitude (m)^(1)^	*alti*
	Mean slope (degrees) (calculated from *Alti*)	*slop*

Water availability	Mean annual precipitation (mm)^(2)^	*prec*
	Mean relative air humidity in January at 07:00 hours (%)^(2)^	*hjan*
	Mean relative air humidity in July at 07:00 hours (%)^(2)^	*hjul*

Environmental energy	Mean annual insolation (hours/year)^(2)^	*inso*
	Mean annual solar radiation (kwh/m^2^/day)^(2)^	*srad*
	Mean temperature in January (°C)^(2)^	*tjan*
	Mean temperature in July (°C)^(2)^	*tjul*
	Mean annual temperature (°C)^(2)^	*temp*
	Mean annual number of frost days (min. temperature ≤ 0°C)^(2)^	*dfro*
	Mean annual potential evapotranspiration (mm)^(2)^	*pet*

Productivity	Mean annual actual evapotranspiration (mm) (=min [*PET*, *Prec*])	*aet*

Environmental disturbance	Maximum precipitation in 24 hours (mm)^(2)^	*mp24*
	Relative maximum precipitation (=*MP24/Prec*)	*rmp*

Climatic variability	Mean annual number of days with precipitation ≥ 0,1 mm^(2)^	*dpre*
	Annual temperature range (°C) (=*TJul*-*TJan*)	*tran*
	Annual relative air humidity range (%) (=|HJan-HJul|)	*hran*

Human activity	Distance to a highway (km)^(3)^	*dhi*
	Distance to a town with more than 100,000 inhabitants (km)^(3)^	*u100*
	Distance to a town with more than 500,000 inhabitants (km)^(3)^	*u500*

**Table 2:** Number of analysed presences and absences and measures of the overall similarity between the predictions produced by modelling combined species distribution data and by fuzzy logic operations between individual species models. For model abbreviations, please see Section 2. Spearman's correlations (with Dutilleul's correction for spatial autocorrelation) were all highly significant (*P* < 0.001).

Model comparison	*N* events	*N* nonevents	Spearman's correlation	Fuzzy numerical comparison
C1 versus C2 (favourability for presence of both)	2412	3052	0.873	0.830
D1 versus D2 (favourability for presence of any)	4273	1191	0.840	0.855
E1 versus E2 (favourability for *B. bufo* instead of *E. calamita*)	1142	719	0.788	0.724
F1 versus F2 (favourability for *E. calamita *instead of *B. bufo*)	719	1142	0.861	0.676

**Table 3:** *Bufo bufo*.

	Estimate	Std. Error	*z* value	*P*
(Intercept)	−0.6473339	0.9170176	−0.706	0.480243
aet	0.0034684	0.0003854	8.999	<2e-16***
slop	0.1376538	0.0200160	6.877	6.10e-12***
d500	−0.0046584	0.0005789	−8.046	8.53e-16***
icon	−0.0278620	0.0103935	−2.681	0.007346**
dhi	0.0087021	0.0015335	5.675	1.39e-08***
prec	−0.0003706	0.0001746	−2.122	0.033810*
temp	−0.2663412	0.0481650	−5.530	3.21e-08***
rmp	1.6677263	0.4787252	3.484	0.000495***
tjan	0.0798147	0.0416233	1.918	0.055168^.^
hjul	0.0239101	0.0057019	4.193	2.75e-05***
dsno	−0.0173457	0.0067807	−2.558	0.010524*
srad	0.3948201	0.1512332	2.611	0.009036**
alti	−0.0005184	0.0002357	−2.200	0.027810*

**Table 4:** *Bufo calamita*.

	Estimate	Std. Error	*z* value	*P*
(Intercept)	−1.4461972	0.5215066	−2.773	0.005552**
Prec	−0.0004168	0.0001974	−2.112	0.034706*
d500	−0.0032900	0.0005710	−5.762	8.33e-09***
Dhi	0.0070846	0.0015043	4.710	2.48e-06***
Aet	0.0028592	0.0004159	6.874	6.24e-12***
d100	−0.0042235	0.0010702	−3.947	7.93e-05***
Alti	0.0003358	0.0001139	2.949	0.003189**
Inso	0.0006156	0.0001559	3.948	7.88e-05***
Rmp	3.9730635	0.8842854	4.493	7.02e-06***
pm24	−0.0055301	0.0016745	−3.302	0.000958***
Pet	−0.0014127	0.0005897	−2.396	0.016584*

**Table 5:** *B. bufo *and *B. calamita*.

	Estimate	Std. Error	*z* value	*P*
(Intercept)	−0.2758076	0.4732180	−0.583	0.56001
d500	−0.0034426	0.0005547	−6.207	5.41e-10***
Dhi	0.0074735	0.0014794	5.052	4.38e-07***
Aet	0.0036445	0.0003296	11.057	<2e-16***
Prec	−0.0009242	0.0001282	−7.211	5.56e-13***
Alti	0.0002249	0.0001106	2.034	0.04197*
Rmp	2.2063901	0.3935660	5.606	2.07e-08***
Pet	−0.0018473	0.0004894	−3.775	0.00016***
d100	−0.0032504	0.0010754	−3.022	0.00251**
Perm	−0.0969069	0.0381119	−2.543	0.01100*

**Table 6:** *B. bufo* or *B. calamita*.

	Estimate	Std. Error	*z* value	*P*
(Intercept)	4.1342959	0.4957333	8.340	<2e-16***
aet	0.0019913	0.0003558	5.597	2.19e-08***
d500	−0.0055607	0.0006259	−8.884	<2e-16***
slop	0.1132909	0.0174535	6.491	8.53e-11***
vtem	−0.0950495	0.0281901	−3.372	0.000747***
dhi	0.0078209	0.0016820	4.650	3.32e-06***
prec	−0.0008682	0.0001658	−5.237	1.64e-07***
temp	−0.1793878	0.0530935	−3.379	0.000728***
tjan	0.1432213	0.0534457	2.680	0.007368**

**Table 7:** *B. bufo* and not *B. calamita*.

	Estimate	Std. Error	*z* value	*P*
(Intercept)	−3.6158776	0.9141552	−3.955	7.64e-05***
prec	0.0014185	0.0003948	3.593	0.000327***
slop	0.1728488	0.0274526	6.296	3.05e-10***
icon	−0.0640986	0.0130270	−4.920	8.63e-07***
d100	0.0083717	0.0019799	4.228	2.35e-05***
hjul	0.0403393	0.0096243	4.191	2.77e-05***
aet	0.0014935	0.0006798	2.197	0.028022*

**Table 8:** *B. calamita* and not *B. bufo*.

	Estimate	Std. Error	*z* value	*P*
(Intercept)	3.6158776	0.9141552	3.955	7.64e-05***
Prec	−0.0014185	0.0003948	−3.593	0.000327***
Slop	−0.1728488	0.0274526	−6.296	3.05e-10***
Icon	0.0640986	0.0130270	4.920	8.63e-07***
d100	−0.0083717	0.0019799	−4.228	2.35e-05***
Hjul	−0.0403393	0.0096243	−4.191	2.77e-05***
Aet	−0.0014935	0.0006798	−2.197	0.028022*

## Appendix

Variables included in each environmental favourability model, their parameter estimates (coefficients) and associated standard error, *z* test, and significance (*P*) values. Variable codes as in Table 1. ****P* < 0.001; ***P* < 0.01; **P* < 0.05; ^.^ *P* < 0.1. (See Tables 3, 4, 5, 6, 7, and 8).
